# Human Pluripotent Stem Cells: A Relevant Model to Identify Pathways Governing Thermogenic Adipocyte Generation

**DOI:** 10.3389/fendo.2019.00932

**Published:** 2020-01-21

**Authors:** Xi Yao, Vincent Dani, Christian Dani

**Affiliations:** Université Côte d'Azur, iBV, UMR CNRS/INSERM, Faculté de Médecine, Nice, France

**Keywords:** human induced pluripotent stem cells, brown adipocytes, adipocyte progenitors, drug discovery, cell-based therapy, obesity

## Abstract

Brown and brown-like adipocytes (BAs) are promising cell targets to counteract obesity thanks to their potential to drain and oxidize circulating glucose and triglycerides. However, the scarcity of BAs in human adults is a major limitation for energy expenditure based therapies. Enhanced characterization of BA progenitor cells (BAPs) and identification of critical pathways regulating their generation and differentiation into mature BAs would be an effective way to increase the BA mass. The identification of molecular mechanisms involved in the generation of thermogenic adipocytes is progressing substantially in mice. Much less is known in humans, thus highlighting the need for an *in vitro* model of human adipocyte development. Pluripotent stem cells (PSCs), i.e., embryonic stem cells and induced pluripotent stem cells, help gain insight into the different phases in the development of multiple cell types. We will discuss the capacity of human PSCs to differentiate into BAs in this review. Several groups, including ours, have reported low spontaneous adipocyte generation from PSCs. However, factors governing the differentiation of induced pluripotent stem cell-derived BA progenitors cells were recently identified, and the TGFβ signaling pathway has a pivotal role. The development of new relevant methods, such as the differentiation of hPSC-BAPs into 3D adipospheres to better mimick the lobular structure of human adipose tissue, will also be discussed. Differentiation of human PSCs into thermogenic adipocytes at high frequency provides an opportunity to characterize new targets for anti-obesity therapy.

## Introduction

The development of obesity and associated metabolic disorders such as diabetes and heart diseases is a major health issue. Obesity results from an imbalance between calorie intake and energy expenditure. The scientific community is focusing attention on white adipose tissue (WAT) that stores energy, and on means to fight its expansion. However, modern lifestyles are often not compatible with a reduction in energy intake. Current anti-obesity drugs to reduce energy intake may have major side effects for the patients. Bariatric surgery has proven efficient for obesity, although long-term complications and obesity relapse may occur. The identification of new anti-obesity targets is thus urgently required. In contrast to WAT, classical brown adipocytes and brown-like adipocytes (BAs) dispersed in WATs, mainly in subcutaneous fat depots, are specialized in energy expenditure thanks to their high content of mitochondria expressing the uncoupling protein-1 (UCP1) (1). Upon activation, BAs consume metabolic substrates and burn fat and sugars via uncoupling of oxidative phosphorylation, in turn inhibiting ATP synthesis (2). The ability of BAs to actively drain circulating glucose and triglycerides to oxidize them can prevent hyperglycemia and hypertriglyceridemia. BAs secrete adipokines that may also contribute to metabolic effects (3). BAs are therefore promising cell targets to counteract obesity and type-2 diabetes. However, major obstacles hamper BA-based treatment of obesity, including the scarcity of BAs in adult humans.

## How to Increase the Mass of Brown-Like Adipocytes in Obese Patients?

Brown adipocytes present at birth persist only around deep organs in healthy adult humans. In addition, BA activity is lower in overweight and obese individuals than in lean ones (4). The proof-of-concept of the beneficial effects of brown fat transplantation has been achieved in rodents, where normoglycemia was restored in diabetic mice and obesity reduced in Ob/Ob mice (5–7). This has given rise to the notion of increasing the BA mass in obese patients as a therapeutic approach to counteract obesity and its associated metabolic complications. A challenge now is to identify an abundant source of human BA progenitors (BAPs) for transplantation. The generation of induced pluripotent stem cells from obese patients as an unlimited source of BAPs that could be expanded for autologous transplantation is a recently discussed option [(8–10) and see below]. Another option that we discuss in the present review is to promote endogenous BA generation in obese patients. Understanding the mechanisms governing the commitment of human pluripotent stem cells toward the brown-like adipogenic lineage, as well as the differentiation of BAPs into functional BAs, should help addressing this issue.

## Human Cell Models Available for Investigating Brown-Like Adipocyte Biology

The identification of molecular mechanisms involved in thermogenic adipocyte generation is progressing substantially in mice. However, much less is known in humans, thus highlighting the need for an *in vitro* model of human adipocyte development. Because of the rareness of BAs in adult humans, immortalized cell lines or multipotent stem cells derived from adipose tissues of young donors (hMADS cells) are the main cellular models used to identify pathways critical for adipogenesis. PAZ6 cells are preadipocytes derived from the vascular stromal fraction of infant BAT which have been immortalized *ex vivo* using the SV40T and t antigens (11). Human preadipocytes from adult BAT localized in deep neck fat can also be immortalized, as recently described (12). hMADS cell lines have been isolated from adipose tissues of young donors in our laboratory. They are not immortalized cells, but can be maintained for several passages *in vitro* thanks to the intrinsic high self-renewal capacity of stem cells (13, 14). Interestingly, hMADS cells can be converted into functional brown-like adipocytes (15). However, the features of infant hMADS dramatically decrease with aging. In addition, these cells are already committed in the adipose lineage, thus precluding the possibility of investigating the earliest steps of adipogenesis.

## Pluripotent Stem Cells Represent a Powerful Model to Identify Pathways Governing Thermogenic Adipocyte Development

Pluripotent stem cells (PSCs), i.e., embryonic stem cells (ESCs) and induced pluripotent stem cells (iPSCs), display a quasi-unlimited self-renewal capacity and are an abundant source of multiple cell types of therapeutic interest. Some papers in the early 2000s reported the potential of human ES cells to generate adipocytes (16–18). These observations suggested that PSCs could be a valuable tool to identify pathways regulating the different steps of adipogenesis, i.e., from the generation of adipose progenitors to their differentiation into mature adipocytes. Then, Taura et al. demonstrated that human iPSCs have an adipogenic potential comparable to that of human ES cells (19). However, these authors did not address the adipogenesis efficiency and the phenotype of adipocytes generated. Surprisingly, a cocktail of hematopoietic factors allowed Nishio and colleagues to report, for the first time, the capacity of human iPSCs to generate substantial BAs (20). These findings support the idea that, as previously shown in mice (21), the BMP signaling pathway plays a critical role in human brown adipocyte generation. However, Nishio did not purify BAPs from differentiating hiPSCs and there was no evidence that the stem cells progressed through a complete adipogenic program to generate adipocytes. Ahfeldt et al. purified hiPSC-derived fibroblasts that were able to undergo differentiation into white adipocytes or BAs following forced expression of adipogenic master genes (22). This strategy allows the generation of human BAs and may be a powerful tool for drug discovery, but the question arises as to whether these cells with ectopic expression of adipogenic master genes faithfully reflect physiological adipogenesis. More recently, a procedure to isolate expandable BAPs from hiPSCs and to generate high levels of functional BAs with no gene transfer was described (8, 23, 24). West and colleagues clonally derived several white- and brown- adipocyte progenitors from hES cell lines and assessed their adipogenic potential when encapsulated in a biocompatible matrix approved for use in human clinical studies (25). These models provide an opportunity to make effective use of hiPSC features to identify critical pathways governing the development of brown-like adipocytes.

## Human Pluripotent Stem Cell Commitment Toward the Brown-Like Adipogenic Lineage is Negatively Regulated by the Retinoic Acid Pathway

**Figure 1:**
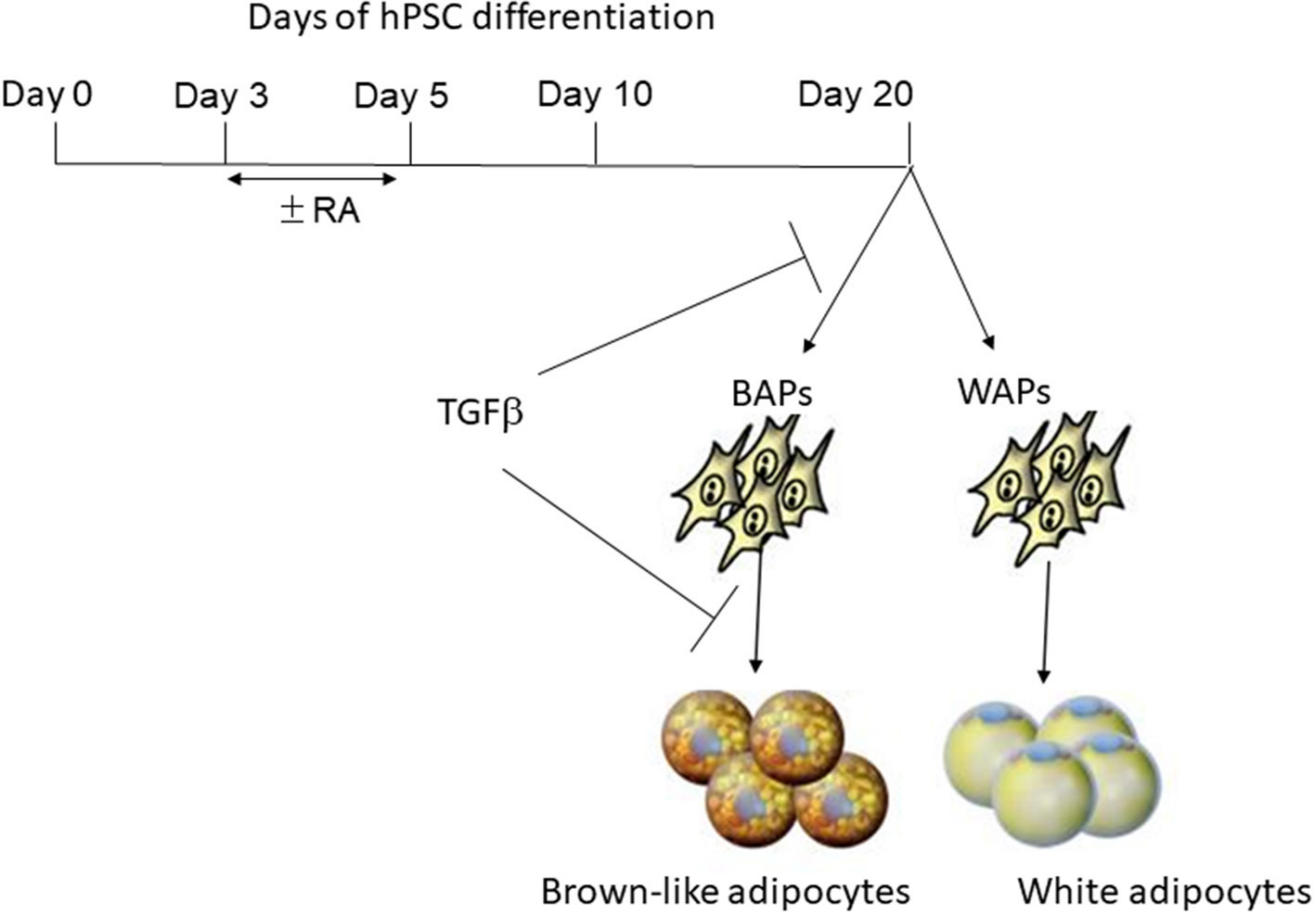
Regulation of brown-like adipose progenitor generation by RA and TGFβ pathways. Treatment of early differentiated hPSCs with retinoic acid (RA) for a short period of time (between days 3–5) inhibits the generation of brown-like adipose progenitors (BAPs) while promoting the generation of white adipose progenitors (WAPS). The TGFβ pathway inhibits both the generation of BAPs and their differentiation into mature adipocytes.

Mohsen-Kanson and colleagues, in our laboratory, investigated factors involved in the commitment of pluripotent stem cells toward adipogenic lineages (23). Four hiPS cell lines and one hES cell line were studied for that purpose. Adipogenic markers, including *UCP1, Dio2, PGC1*α, and *PRDM16*, were detected in differentiated cultures, indicating that cells having a brown-like adipocyte gene program were spontaneously generated during differentiation. However, the adipogenesis efficiency was weak. Indeed, adipocytes were co-stained with LipidTox (for triglyceride staining) and CD73 (an adipocyte cell surface marker), and then quantified by flow cytometry (26). The data showed that the number of LipidTox^+^/CD73^+^ cells represented only 2% of cells in the differentiated cultures. Small-scale drug screening to uncover signaling pathways regulating the earliest steps of human adipogenesis revealed that the retinoic acid (RA) pathway promoted hiPSCS commitment toward the adipogenic lineage by increasing the number of LipidTox^+^/CD73^+^ cells to 15%. In contrast, expression of the brown adipocyte specific marker *UCP1* was inhibited in RA-treated cultures. Together, these data support the hypothesis that RA pathway activation at an early development stage dramatically promotes the differentiation of human PSCs into the UCP1-negative adipocyte lineage, while inhibiting UCP1-positive adipocyte generation (see Figure 1). This observation is reminiscent of the critical role of RA in the early steps of mouse ES cell white adipogenesis (27, 28). The identification of RA targets could provide a means to uncover genes involved in the earliest steps of adipogenesis. The combination of computational and experimental approaches in mouse ES cells revealed an extensive network of transcription factors that might coordinate the expression of genes essential for the acquisition of adipocyte characteristics (29). This could represent a unique comprehensive resource that could be further explored to investigate human adipocye development.

## Critical Role of the TGFβ Pathway in hiPSC-BA Progenitor Differentiation

Several research groups, including ours, have reported that hiPSC-BAPs display a low adipogenic capacity that hamper their use in cell-based therapy and basic research. In fact, Chen et al. first underlined the limited capacity of hiPSC-derived progenitors to undergo adipocyte differentiation, a feature that is often observed by authors but not always pointed out (30). Interestingly, the low adipogenic differentiation potential is not restricted to hiPSC-derived cells as cells derived from human hESCs display the same feature, thus ruling out the possibility that the low hiPSC-adipogenic capacity could be due to incomplete reprogramming. The low adipogenic potential of adipose progenitors is also not dependent on the methods used to derive them from PSCs (31). Overall, these observations indicated that appropriate culture conditions had to be set up to unlock hPSC-BAP adipogenesis. Ascorbic acid, EGF, hydrocortisone, activin A and IL4 have been shown to regulate hiPSC-BAPs differentiation (8, 23, 24, 32) (see Table 1). However, TGFβ signaling has a pivotal role. The TGFβ pathway has emerged as a critical anti-adipogenic player through the Smad 2/3 activation (33–35). Deletion of TGFβ receptor 1 in mice has been shown to promote brown-like adipogenesis within white adipose tissue, thus supporting a model where TGFβ receptor signaling plays a role in regulating the pool of BAPs (36). The Smad2/3 pathway was found to be active during hiPSC-BAP differentiation, suggesting that bioactive TGFβ family members were secreted, which might lock differentiation (24). In agreement with this hypothesis, Su et al. showed more recently that the expression of TGFβ-ligands and -receptors increased during the differentiation of FOXF1 mesoderm progenitors toward adipocytes during hiPSCs development (8). The anti-adipogenic role of the TGFβ pathway has also been functionally demonstrated via use of the TGFβ inhibitor SB431542 (37). Inhibition of the active Smad 2/3 pathway upon SB431542 addition during hiPSC-BAP differentiation induced a sharp increase in *UCP1* expression and in the number of mature BAs (8, 24, 38). Figure 1 illustrates the regulation of brown-like adipocyte generation from hPSCs by the retinoic acid and TGFβ pathways. Wankhade and colleagues proposed that negative regulation of PGE2/Cox-2 by TGFβ is involved in the recruitment of brown-like adipose progenitors (36). Interestingly, inhibition of the TGFβ pathway is not a prerequisite for adult adipose tissue-derived adipose progenitor differentiation. The low hiPSC-BAP adipogenic capacity compared to human adult-BAPs is reminiscent of the findings of Wang et al. who described distinct mechanisms regulating differentiation of embryonic-like and adult adipose progenitors in mice (39). Our hypothesis is that several pathways inhibit the development of PSC-BAs, which means that small molecules must be used to unlock differentiation. We also hypothesize that the current 2D culture conditions are not effective in promoting hiPSC-BAP differentiation. The development of new *in vitro* culture methods better mimicking the structure of human adipose tissue could now help decipher relevant regulators of BA adipogenesis.

**Table 1:** Pathways regulating brown/brown-like adipocyte generation from human pluripotent stem cells.

**hPSCs**	**Pathways/facors**	**References**
hESCs, hiPSCs	BMP7, VEGFA, FLT3LG, IL6, IGF2	Nishio et al. (20)
hESCs, hiPSCs	Retinoic acid	Mohsen-Kanson et al. (24)
hiPSCs	BMP7, activin A	Guenantin et al. (32)
hiPSCs	Ascorbic acid, hydrocortisone, EGF	Hafner et al. (38)
hiPSCs	IL4	Su et al. (8)
hiPSCs	TGFβ	Hafner et al. (38) Su et al. (8)

## The Next Steps Toward Gaining Greater Insight Into the Development of Human BAs: 3D Adipospheres Generation

The weak efficacy of hiPSC-BAP differentiation might partially be explained by the culture conditions, which do not mimic the physiological microenvironment in which cells normally reside within adipose tissue. Cells are conventionally grown as monolayers in 2D, which is out of line with the *in vivo* situation. Adipose tissue exhibits a complex lobular architecture that plays a functional role in adipogenesis (40). Indeed, adipose tissue is highly vascularized and made up of lobules, corresponding to clusters of adipocytes, separated from each other by a structured extracellular matrix (41). Interestingly, it has been proposed that the adipocyte browning phenomenon specifically occurs in these lobules (42). In an effort to enhanced the physiological relevance of *in vitro* studies, 3D culture technologies and bioengineering methods for seeding different cell types in an organoid-like structure are highly promising (43–46). 3D cultures represent a bridge between traditional cell culture and live tissue. But, could these new technologies be applied to hiPSC-BAPs maintenance and differentiation? It is interesting to note that hiPSC-BAs have been generated by several teams via the formation of hiPSC-embryoids in suspension (20, 22, 23, 38). This strongly suggests that hiPSC-BAPs could be maintained in 3D culture conditions. The next challenge will be the generation of 3D hiPSC-brown-like adipospheres resembling human adipose tissue. The challenges will include to enrich hiPSC-brown-like adipospheres with endothelial cells and macrophages embedded in an extracellular matrix allowing functional bidirectional interactions between the microenvironment and adipocytes.

## Conclusions

Human pluripotent stem cells provide an opportunity to characterize pathways that play a role in the different steps of thermogenic adipocyte development. Some factors have been identified, but their impact on other hiPSC-derived cells of interest such as white adipocytes, endothelial cells and macrophages, has yet to be determined and integrated in a relevant model. 3D culture of hiPSC-adipospheres in which BAs interact with cell types that are present in the adipose tissue microenvironment will provide a more suitable physiological *in vitro* condition that should lead to the identification of druggable pathways to counteract obesity and its associated metabolic disorders.

## Author Contributions

XY, VD, and CD contributed to the conception and design of the review. XY and VD generated the data shown. CD wrote the manuscript. All authors approved the submitted version.

### Conflict of Interest

The authors declare that the research was conducted in the absence of any commercial or financial relationships that could be construed as a potential conflict of interest.
